# Supplementary material for: Are carbon emissions trading and green financial instruments synergistic? -Comprehensive quantitative research based on content analysis
Source: PLoS One. 2024 Mar 7;19(3):e0298601. doi: 10.1371/journal.pone.0298601 (PMC10919601; doi:10.1371/journal.pone.0298601)
Supplement: S1 Data — (ZIP) [file pone.0298601.s001.zip › Data/地级市Z_F工作报告.zip!/║┌┴·╜¡/╣■╢√▒⌡╩╨/└┤╫╘╣1⁄2╓┌║┼í╛┬φ┐╦╩2╛▌═°í┐_2012.docx]

各位代表：

　　现在，我代表市人民政府向大会报告工作，请予审议，并请市政协各位委员提出意见。

　　过去五年工作回顾

　　2007年以来的五年是极不平凡的五年，是我们抢抓机遇、应对挑战、推动经济社会更好更快发展的五年，是超越自我、敢于担当、迎难而上、开拓进取的五年。五年来，在省委、省政府和市委的正确领导下，市政府紧紧依靠全市人民，深入贯彻落实科学发展观，奋发图强，真抓实干，圆满完成十三届人大确定的各项任务。

　　经济实力显著增强。地区生产总值跨越两个千亿元台阶，2011年达到4243.4亿元，比上年增长12.3%，五年年均增长13.2%。人均地区生产总值超过4万元，比上年增长12%，年均增长12.8%。财政收入大幅增加，财政一般预算总收入完成502.1亿元，比上年增长22.3%，是2006年的2.6倍。其中地方财政一般预算收入完成300.3亿元，增长30.5%，是2006年的2.5倍。金融、信息、会展、物流等现代服务业加快发展，服务业增加值达到2149亿元。累计实现社会消费品零售总额7648.4亿元，年均增长18.3%。产业结构更趋合理，三次产业结构由2006年的14.1：35.8：50.1调整为10.5：38.8：50.7。非公有制经济增加值在地区生产总值中占据“半壁江山”。科技进步对经济增长贡献率由44.6%提高到54.4%。万元GDP能耗累计下降21.5%，水、气主要污染物排放分别减少17.8%和11.7%。

　　人民生活明显改善。惠民力度持续加大，向市民承诺的280个惠民行动项目件件落实，累计投入871.5亿元。城乡居民收入持续提高，2011年城市居民人均可支配收入突破2万元，比上年增长14.1%，五年年均增长12.3%；农村居民人均纯收入9608元，比上年增长19.8%，是2006年的2.18倍。实施积极的就业、创业政策，累计新增城镇就业53.8万人，登记失业率控制在3.3%以内，年转移农村劳动力120万人以上。社会保障覆盖面持续扩大，保障标准大幅提高，困难群体得到政府救助和社会关爱，养老服务设施进一步完善。主城区居民全部饮用磨盘山水，103万户居民用上清洁安全天然气，集中供热普及率由61%提高到72%，城市生活垃圾无害化处理率由54%提高到75%，城区生活污水集中处理从无到有，处理率达80%。累计更新公交车辆3200余台，开辟调整公交线路74条,农村客运班车通村率达100%。

　　社会事业全面进步。各级各类教育全面发展，教育公平进一步显现，累计创建标准化学校400余所、资助贫困学生62万人次。医药卫生体制改革扎实推进，新农合统筹区域内住院政策性报销比例达70%，社区卫生服务机构基本实现全覆盖，251个政府办基层医疗卫生机构实行基本药物零差率销售。人口自然增长率为3.2％。。文化建设取得新进展，覆盖全市的公共文化服务网络基本形成，哈尔滨大剧院等标志性文化设施加快建设，中华巴洛克历史文化街区保护开发等项目进展顺利，被联合国授予“音乐之城”称号。群众体育事业蓬勃发展，竞技体育水平进一步提高。安装健身路径6500件。成功举办第24届世界大学生冬季运动会。

　　改革开放持续深化。经济体制改革取得重大进展，国有企业改制基本完成，厂办大集体改革接近收尾，国有净资产总额比2006年翻两番。农村产权确权登记颁证任务完成80%。市政府组成部门由49个精简到42个，下放和取消管理权限85项。财税、金融、文化、水务等领域改革取得明显成效。对外开放进一步扩大，累计实现进出口总额189.2亿美元，年均增长14.9%。累计实际使用外资30.9亿美元、国内区域经济合作资金到位2063.7亿元，年均分别增长16.4%和34.2%。“哈洽会”、“冰洽会”等招商平台作用凸显，华南城、国际农业博览中心等102个招商大项目实现当年签约、当年落地。摩根大通、东亚银行等国际知名银行入驻我市。哈尔滨与以色列合作取得突破，“哈以基金”在国企战略投资、科技企业孵化等方面发挥积极作用。

　　精神文明与民主法制建设得到加强。“三优”文明城市和全国文明城市创建工作进展明显，市民文明素质进一步提升。荣获全国双拥模范城“七连冠”。自觉接受人大法律监督和政协民主监督，人大代表建议、政协委员提案办复率均达100%。提请市人大常委会审议地方性法规草案24件，制定政府规章92件。“五五”普法圆满完成。社会管理成效明显，社区工作用房和居民公益性服务设施不断完善，形成了社区网格化管理和“中心社区”建设模式。各类生产安全事故下降61.5%，信访形势有所好转，万人刑事发案率在副省级城市中保持最低。

　　新闻出版、广播电视、民族宗教、外事侨务、人力资源、统计、审计、气象、人防、地震、档案、国防教育和双拥及民兵预备役、地方志、老龄、残疾人等工作取得新进步。

　　各位代表！五年来，特别是市委确定“北跃、南拓、中兴、强县”发展战略以来，我们认真组织、强力推进，哈尔滨发生了一系列重大变化：

　　一是城市空间迅速拓展，“一江居中、两岸繁荣”迈出新步伐。通过建设北国水城、科技创新城、工业新城、群力、哈西、哈东等新城区，实现了城市发展空间向北跨越、向南拓展、向县域延伸，使哈尔滨发展空间更加广阔。新一轮《城市总体规划（2011－2020）》获得国家批复。科技创新城累计开发9.6平方公里，134个研发项目入驻园区，37个产业项目和配套项目开工建设。工业新城开发35平方公里，新增规模以上企业93户，新引进世界500强企业11家。宾西、利民开发区晋升为国家级开发区，哈西、群力成为新的投资热点和宜居新区。

　　二是大项目建设快速推进，固定资产投资大幅增长。预计2011年实现固定资产投资3012亿元，是2006年的3.7倍。五年累计实施亿元以上大项目1833个，完成投资3788亿元。工业投资大幅增长，累计完成2635亿元，年均增长34.9%，投资增幅连续4年居15个副省级城市首位，总量排名由第14位晋升至第8位。民间投资拉动力增强，累计完成4759.3亿元，年均增长33%，占全部投资比重由47%提高到50.3%。

　　三是产业结构优化升级，工业经济持续提速增效。2011年规模以上工业增加值比上年增长14%，五年年均增长15%。规模以上工业企业利税由100亿元提高到260亿元以上，年均增速超过21%。装备制造、食品、医药和化工四大主导产业占规模以上工业比重由75.7%提高到83.1%。以民用航空、生物、新材料、新能源装备、电子信息、绿色食品六大战略性新兴产业为代表的高新技术产业快速发展，产值由876亿元增加到2452亿元，年均增长22.9%。

　　四是强县战略深入推进，县域经济平稳快速发展。2011年县域财政一般预算收入实现41亿元，年均增长30.3%。县域工业主导地位更加突出，双城食品、五常医药、依兰煤化工等特色产业加快发展。农业综合生产能力明显提高，粮食产量突破280亿斤，“旱改水”、“粮改菜”和“粮牧换位”成效显著。农田水利建设不断加强，水资源供给和防洪抗旱减灾能力持续提高。

　　五是冰雪和湿地旅游引领作用突出，旅游业对经济增长拉动力增强。冰雪旅游持续上层次、上规模，哈尔滨冰雪节已成为国际冰雪旅游盛会，连续五年入选“全国冬季最热旅游城市”。立足保护开发利用松花江湿地资源，制定出台了《万顷松江湿地保护利用规划》，形成了以旅游业为牵动、服务业配套发展、实实在在增加百姓收入的新的产业体系，成功举办首届“中国·哈尔滨松花江湿地旅游文化节”，哈尔滨夏季旅游迅速升温，成为全国“十大避暑旅游城市”。2011年旅游总收入完成457.8亿元，比上年增长20%，是2006年的2.7倍，占GDP比重由8.2%提高到11.5%。

　　六是重大基础设施建设取得突破，城乡载体功能显著提升。基础设施投入力度前所未有，累计完成投资2700亿元以上。路桥建设实现历史性突破，新改建各类道路667条、桥涵121座，维修道路3686条（段）。松浦大桥、松花江公路大桥西桥、阳明滩大桥相继建成通车，依兰松花江大桥顺利推进。新建哈阿快速干道，打通长江路，拓宽改造哈五路、哈双北路、东巨路等出城口道路，绕城高速全线建成，中山路、机场路、文昌高架桥、征仪跨线桥等交通干道贯通城区，地铁1号线、哈西客站等工程进展顺利，城市立体交通网络初步形成。乡镇全部实现高等级公路连接，公路通村率达100%。

　　七是环境保护和综合整治得到加强，城乡面貌明显改善。启动了松花江哈尔滨段污染治理，干流出境水质主要污染物下降21%。“三沟一河”整治取得成果，马家沟、何家沟、信义沟基本实现污水全线收集和集中处理，阿什河清淤工程全面展开，部分城区段实现污水截流。完成双琦废弃资源综合利用示范一期工程。累计拆炉并网、限期治理超标排放燃煤锅炉3360台。环卫作业投入大幅增加，城区道路清扫和清冰雪机械化率、水冲洗率明显提高。开展环境综合整治“三大战役”，城乡卫生环境和市容面貌显著改观。新农村建设扎实推进，五年投入资金超过200亿元。累计造林208万亩，森林覆盖率达45.5%。

　　八是保障性安居工程建设力度加大，困难群众住房条件极大改善。棚户区和农村泥草房改造取得重大进展，累计拆迁改造棚户区1235万平方米、农村泥草房1200万平方米。建设各类保障性住房9.9万套，发放经济适用住房货币补贴2.4亿元、廉租住房补贴1.9亿元，实物配租廉租住房8777套。公积金用于保障房建设资金22亿元。农村住房砖瓦化率由78.9%提高到89.3%。群众居住条件明显改善，财产性收入大幅增加。

　　九是广大干部群众干事创业氛围日益浓厚，城市凝聚力和吸引力明显增强。新战略凝聚人心、汇集力量，充分调动了广大干部群众谋发展、惠民生的积极性和创造性。党风廉政建设责任制深入落实，“政风建设年”成果明显，“超越自我、执政为民”深入人心，迎难而上、敢于担当成为工作主流，行政效能和服务质量得到较大提高，经济发展环境进一步改善。“创业创新、全民成才”氛围初步形成。被国家确定为首批创新型试点城市、创业型试点城市、现代服务业综合改革试点区域、水生态修复与保护试点城市、电子商务示范城市。

　　这些成绩的取得，是省委、省政府和市委坚强领导、科学决策的结果，是全市广大干部群众团结一心、拼搏奋进的结果，是人大代表、政协委员及社会各界人士鼎力支持、共同努力的结果，也是历届政府扎实工作、奠定坚实基础的结果。在此，我代表市人民政府，向全市各族人民、各民主党派、工商联和无党派人士，向驻哈部队、武警官兵和中省直驻哈机关企事业单位，向关心、支持哈尔滨发展的港澳台同胞、海外侨胞和国际友人，致以崇高的敬意和衷心的感谢！

　　在肯定成绩的同时，我们也清醒地认识到，我市经济社会发展仍然面临许多矛盾，工作中还存在不少问题：一是经济总量小、增速慢仍然是我市的主要矛盾，加快发展、壮大实力、增强竞争力的要求非常迫切。二是产业结构不尽合理，做强工业、提升农业、发展服务业的力度仍需加大。三是县域经济实力较弱，农村发展相对落后，统筹城乡发展、破解“三农”问题的任务依然艰巨。四是影响科学发展的体制机制障碍仍然没有完全消除，非公有制经济发育不充分、开放度偏低的问题还没有得到根本解决。五是城市功能还不完备，基础设施依然薄弱，城市管理水平和运行质量有待进一步提升。六是城乡居民收入增长缓慢，社会保障水平还不高，社会事业发展相对滞后，改善民生的任务还十分繁重。七是环境保护、生产安全、食品安全等方面的矛盾仍比较突出。八是政府职能转变还不到位，少数政府工作人员责任意识、服务意识和危机感、紧迫感不强，办事效率不高、抓落实不到位的问题还不同程度存在，铺张浪费和腐败现象在一些地方和领域还时有发生。这些问题，我们将高度重视，在今后工作中认真解决。

　　今后五年及2012年工作建议

　　刚刚闭幕的市第十三次党代会，在总结过去五年成就、科学分析当前和今后一个时期形势的基础上，绘就了“坚定实施新战略、奋力实现哈尔滨科学发展新跨越”的宏伟蓝图。面对新形势、新任务、新要求，我们必须全面贯彻落实十三次党代会精神，进一步抢抓东北老工业基地振兴历史机遇，凝神聚力谋发展，千方百计惠民生，努力建设繁荣幸福文明和谐的哈尔滨，在全省“八大经济区”、“十大工程”建设中更好地发挥龙头带动作用。今后五年政府工作指导思想是：高举中国特色社会主义伟大旗帜，以邓小平理论和“三个代表”重要思想为指导，深入贯彻落实科学发展观，按照党中央、国务院、省委、省政府和市委的决策部署，进一步超越自我，坚定不移实施“北跃、南拓、中兴、强县”战略，更加注重保障和改善民生，加快转方式、调结构步伐，深化改革开放创新，统筹城乡建设发展，提升城市文明形象，维护社会和谐稳定，全面推进经济、文化、社会和生态建设，团结一心、乘势而上，为实现哈尔滨科学发展新跨越而奋斗。

　　未来五年实现科学发展新跨越的“四大形象标志”是：老城展现欧陆风情新貌、新城拔地而起、城乡一体化明显进步、经济结构调整取得实质性进展。提升群众福祉的“四大民生指标”是：人均收入翻一番、基本解决住房问题、社会公共服务达到新水平、多年积累的群体性突出矛盾基本解决。按照这个目标要求，今后五年我们要：

　　———着力实施惠民工程，努力让群众生活得更方便更满意更幸福。保障和改善民生是政府一切工作的出发点和落脚点，更是满足人民日益增长物质文化需要的必然要求。针对民生领域群众反映强烈的突出问题，我们将进一步加大财政投入力度，持续推进惠民行动，切实解决老百姓关注的大事、要事、急事、难事，真正让老百姓得到实惠。力争到2016年，城乡居民收入大幅增长，分别达到4万元和2万元左右，比2011年翻一番，最低工资标准年均增长15%左右。新建各类保障性安居工程12万套，人均住房建筑面积15平方米以下的低收入住房困难家庭实现应保尽保，城区四环路内棚户区改造、农村泥草房改造全面完成。累计新增城镇就业55万人以上，登记失业率控制在4.5%以内。百万农民工技能培训工程取得实效，提前实现农民增收100亿元目标。社会养老保险、基本医疗保险和社会救助实现城乡全覆盖。城区和县镇义务教育学校基本达到标准化，学前三年教育毛入园率达到75%。二级甲等以上医院、城区标准化社区卫生服务中心、达标乡镇卫生院和村卫生所基本实现全覆盖。加大投入力度，文化体育设施遍布城乡社区。

　　———着力推进现代化、国际化大城市建设，努力提升城市承载能力。建设现代化、国际化大城市是省委提出的要求，更是哈尔滨未来的发展方向。经过长期的建设和发展，哈尔滨建设现代化、国际化大城市有基础、有潜力，也有现实差距。我们必须在拉开城市骨架的基础上，高起点、高标准推进一批功能性强、承载力大的基础设施项目。持续提升区域性交通枢纽功能，重点抢抓太平国际机场扩建契机，规划建设空港物流经济区。切实做好哈大客专、哈齐客专配套工程建设，力促哈佳、哈牡高速铁路开工建设，确保哈西客站按期建成投入使用，加快改造哈尔滨既有火车站，建成松北火车站，加快推进大功率机车检修基地和集装箱中心站建设，完成内陆港搬迁扩建，同步推进综合保税区建设。力争五年后，基本形成服务全省、辐射东北、联通国内外的大流通格局。持续提升城市道路交通和配套能力，开通地铁1号线，启动建设地铁2号线、3号线；加快推进新滨洲桥、滨北公铁两用桥等跨江通道建设，拓宽打通一批城市主次干道，配套建设松北、哈南、群力、哈西、哈东等新区市政道路。推进北国水城建设，建成江北百公里滨江景观大道，松江避暑城建设取得明显进展。加快水、电、气、热管网升级改造，完善备用水源、气源、热源，确保城市拓展和日常运行需求，城市污水集中处理率、集中供热普及率、生活垃圾无害化处理率分别提高到85%、91%和85%以上。持续提升城市产业服务功能，推进城市综合体、高端商务区等服务设施建设，确保华南城、国际农业博览中心等一批重大项目建成投入使用，为集聚人流、物流、信息流，扩大国际知名度和影响力打下基础。

　　———着力推进产业项目建设，增强经济综合实力和竞争力。产业项目是经济发展的强力支撑，是加快新型工业化进程的根本所在。必须紧紧抓住产业项目建设不放松，深入实施产业倍增计划，大力发展实体经济，重构现代产业体系。通过高新技术改造、资本市场运作、信息化武装、现代化管理等手段，大幅提升传统优势产业支撑力，四大主导产业增加值年均增长16%。强化政策支持和规划引导，大幅提升战略性新兴产业竞争力，尽快将其培育成先导性、支柱性产业，高新技术产业增加值年均增长20%以上。大力发展总部经济、金融保险、会展经济、楼宇经济、社区服务等新兴业态，打造现代服务业集聚区和产业园区。旅游总收入年均增长20%以上，金融、物流产业增加值年均分别增长30%和16%。充分利用哈尔滨适合发展云计算有利条件，加快引进并做大云计算企业，争取成为国家云计算试点城市，力争在国际国内分工布局中占有重要位置。抓好大园区建设，科技创新城研发区已签约开工的100多个项目，产业区LED等高科技项目，确保在三、五年内建成投用，销售收入超过千亿元。工业新城重点发展信息产业、新型装备制造业，确保五年建设100平方公里，新增产值2000亿元以上。利民开发区建成医药产业园，宾西开发区实现加快发展。力争通过五年努力，基本形成结构优化、技术先进、清洁安全、附加值高、吸纳就业能力强的现代产业体系，地区生产总值比“十一五”期末翻一番，财政收入翻一番以上。

　　———着力推进现代农业大发展，加快城乡一体化进程。发展现代农业是转变经济发展方式的重要任务，是经济社会发展的物质基础。必须坚定不移地走现代化大农业发展之路，加大强农惠农富农力度，加快水利化、科技化、机械化、合作化、产业化、市场化、城镇化、生态化建设，确保农业增产、农民增收、农村发展。力争到2016年，现代化大农业建设取得积极进展，粮食生产能力超过300亿斤。县域工业化水平显著提高，县域工业增加值年均增长16%以上。农业产业化进程加快，农产品由初级加工向精深加工延伸、向配套扩张，粮食由食品加工向化工产品加工拓展。现代畜牧业加快发展，畜牧业对经济增长、农民增收的牵动力持续提高。农村城镇化稳步推进，一批特色突出的小城镇初步建成，户籍制度改革取得突破，提前实现百万农民进城镇。

　　———着力加快文化名城建设，提升城市文化软实力。文化大发展大繁荣是满足人民多样化精神文化需求的重要途径，是城市生存发展和振兴繁荣的本质性力量。必须紧紧抓住中央和省委力促文化产业大开发的大好时机，加快文化名城建设。坚持先进文化的前进方向，充分发挥文化引领社会、教育人民、推动发展的重要作用，大力推动社会公德、职业道德、家庭美德、个人品德建设。深入实施“三优”文明城市工程，广泛开展群众性精神文明创建活动和志愿服务活动，明显提升全市人民文明素质。凸显城市文化特色，彰显大气、神气、洋气城市风格，着力打造具有鲜明特征和时代气息的特色文化品牌。把“哈夏”音乐会打造成全国一流和国际性的音乐盛会。扶持文化创意、数字出版、移动多媒体、动漫游戏等新兴文化产业，做大做强书刊发行、影视制作、印刷出版、演艺娱乐等传统文化产业，力争到2016年，文化产业占GDP比重达6%。把平房动漫产业园打造成国内一流的动漫网络游戏产业聚集地，群力文化产业园打造成全省规模最大的文化艺术品贸易园区，哈师大现代文化艺术产业园打造成全市新的文化艺术教育培训中心和文化创意产业研发中心。发展公益文化事业，确保哈尔滨大剧院、音乐厅、城乡规划展览馆、职工文化中心等文化基础设施建成并良性运营，区县（市）文化馆、图书馆达到国家标准，乡镇文化站、农家书屋基本实现全覆盖。

　　———着力深化改革、扩大开放，努力构筑有助于科学发展的体制机制。体制机制不活、开放度偏低是制约哈尔滨发展的最大瓶颈。必须以更大决心和气力推进改革开放，着力解决影响经济长期健康发展的体制性、结构性矛盾，在一些重点领域和关键环节取得新的突破。力争用五年时间，国有企业基本建立现代企业制度，厂办大集体企业改革全面完成。探索建立多元投资主体和多种投资方式的新型投融资体制，筹建文化、旅游、环保、医药等产业投融资平台，建设有地区优势、联接全国的综合性产权要素市场，建立区域股权交易市场，发展大宗物资交易市场及期货市场。新增上市企业15至20户。加快农村综合改革，逐步确立现代农村产权制度。推进政企分开、政事分开，减少政府对微观经济活动的直接干预。完成新一轮事业单位改革，推进经营性事业单位转企改制。启动“三供一业”试点，加快城市供水、供热、供气和物业管理改革。发展非公有制经济，力争非公经济比重超过60%。扩大对外开放，着力在黑龙江和内蒙古东部沿边开发开放中发挥作用，打造对俄开放枢纽，争创中俄合作示范城市。力争市属外贸进出口总额、实际使用外资年均分别增长15%和10%以上，国内区域经济合作资金到位额年均增长20%以上。

　　各位代表！2012年是实施“十二五”规划承上启下的重要一年，也是新一届市政府的开局起步之年，做好今年工作意义重大。按照中央经济工作会议精神和省委经济工作会议提出的力求在提高质量、增强效益上有新突破，在调整结构、转变方式上有新举措，在深化改革、扩大开放上有新作为，在备足后劲、打牢基础上有新进展，在改善民生、促进和谐上有新成效的总体要求，结合落实“十二五”规划，今年主要预期目标是：地区生产总值增长12%左右；地方财政一般预算收入增长15%以上；全社会固定资产投资增长30%以上；规模以上工业增加值增长14%以上；社会消费品零售总额增长15%以上；市属外贸进出口总额增长15%以上；城市居民人均可支配收入增长12%以上；农民人均纯收入增长12%以上；城镇登记失业率控制在4.3%以内；人口自然增长率控制在5.8％。以内；万元生产总值综合能耗降低3.6%；主要污染物化学需氧量和氨氮、二氧化硫、氮氧化物排放总量分别比2010年削减2%和4%、0.4%、0.74%。重点抓好八项工作：

　　（一）以结构调整为主线，进一步壮大工业经济综合实力

　　作为老工业基地城市，结构调整的着力点必须放在工业经济转型升级上。工业经济上不去，哈尔滨就会失去竞争优势，全面振兴就无法实现。必须一手抓传统产业升级改造，一手抓战略性新兴产业加快发展，着力构建新型工业化体系。今年重点抓好哈药总厂优化调整改造、东轻超大规格铝板带材等重大技改项目100个，启动实施兵装集团地源热泵、富霖芯片等新兴产业项目100个。全年实施亿元以上项目200个，投达产项目50个，新增销售收入150亿元。

　　抓好大工业园区建设。园区“小而散”是影响我市项目落地、产业集聚的一大弊端。必须坚持布局合理、功能明确、用地节约、要素集聚，加快园区整合步伐，在推进26个市级产业园区建设的同时，率先发展经开区、高新区、宾西、利民4个国家级开发区，力争产业集中度达到50%以上。工业新城今年确保50个项目开工建设、60个项目签约入驻，完成投资200亿元。科技创新城新开发4平方公里，完成投资50亿元，确保新开工项目面积80万平方米，50个项目竣工投产。利民开发区重点抓好生物医药产业园区建设，力争竣工投产项目10个,完成投资20亿元。宾西开发区力争20个亿元以上项目竣工投产，完成投资40亿元。

　　做大做强骨干企业。加大对哈电、哈飞、东安、东轻等中直省属企业服务力度，为其更好更快发展创造条件。健全重点企业联系机制，将规模以上企业全部纳入服务和监控范围。充分发挥新型工业化资金导向作用，促进要素资源向优势企业集聚。深入实施“骨干企业扶持工程”，推进国有企业战略重组，培育和打造一批带动作用大、支撑能力强的龙头企业，力争年产值10亿元以上企业达45户，规模以上企业1000户。培育名牌产品，努力提升企业核心竞争力。

　　加大节能减排力度。抓好重点领域和企业节能降耗，将监控范围扩大到年耗能5000吨标准煤以上用能企业。落实固定资产投资项目节能评估审查制度和重污染行业退出机制，推行合同能源管理。依法淘汰水泥、造纸等行业落后产能。抓好锅炉窑炉、电机系统等重大节能项目10个，实施中煤龙化等重点脱硫脱硝技术改造工程28项。

　　（二）以现代农业建设为基础，进一步促进农业稳定发展、农民持续增收

　　毫不动摇地抓好粮食生产。越是形势好，我们越要牢固树立抗灾夺丰收的思想，千方百计确保粮食产能提高。加强农业基础建设，重点实施市郊高标准基本农田整治示范项目，确保整理土地33.5万亩，新增耕地4800亩，新建基本农田21.5万亩。完善农田水利设施，完成松花江干流及其一级支流重点堤防险工弱段和10座病险水库消险加固任务，启动运粮河及阿什河东风镇堤防建设。提高现代化大农机装备能力，农业综合机械化程度提高到85%，农机装备产业基地力争实现产值18亿元。加快农业科技创新和新技术推广，提高科教兴农水平。优化种植业结构，增水稻、稳玉米、减大豆，保证主要农作物种植面积。继续推进“旱改水”扩稻工程，新建水稻节水核心示范区14处，新增水田80万亩。落实“一圈两带”蔬菜产业发展规划，重点搞好五常万亩设施蔬菜园区建设。

　　坚持把发展农业产业化作为提高县域工业化水平的关键举措。尊重市场经济规律，拓展农业市场需求和增收空间，引导和服务农民直面市场，把利润更多地留给农民。做大做强稻米、乳品等10大产业链，新建续建投资亿元以上绿色食品产业项目12个，市级龙头企业增加到130家。农业产业化带动基地面积2200万亩以上，参与农户92万户。加快发展现代畜牧业，突出抓好10大牧业龙头企业和30个生产基地建设，引进投资超亿元畜产品加工企业5个。加强合作社、协会、经纪人队伍建设，提高农民组织化水平。

　　稳步推进农村城镇化。坚持城镇化与新农村建设相结合，深入实施“百镇提档升级、二十镇试点引领、六镇示范样板工程”，重点加快双城、宾县等卫星城和五常、尚志等次中心城市建设，抓好新发、团结等国家级小城镇试点。提高农民创业致富本领，完成农民工技能培训15万人，转移农村劳动力138万人。加快新农村建设，建成示范村20个，建设高标准绿化村屯100个。落实中央扶贫工作会议精神和新十年扶贫开发纲要，加大扶贫力度，全面推进新一轮65个村整村扶贫开发。新建农村饮水安全工程433处，解决33.2万人饮水安全问题。实施大中型沼气项目4个、年产万吨秸秆固化项目5处。

　　（三）以现代服务业为引领，进一步推动服务业提档升级

　　旅游业是拉动服务业加快发展、增加财政和群众收入的朝阳产业，更是哈尔滨新一轮加快发展的优势和潜力所在。我们要抢抓中俄两国互办“旅游年”活动契机，进一步做好“冰、雪、山、水”旅游文章，全面提升“冰城夏都”旅游品牌。冰雪游重点启动四季冰雪乐园等新项目，提档升级冰雪大世界等传统项目。湿地游重点推进道外滨江湿地、太阳岛东部湿地旅游景区、狗岛湿地休闲运动场和伏尔加庄园二期等项目，加快发展赛艇、帆船等高层次旅游项目，启动松江避暑城起步区建设。深度挖掘现有国家级森林公园以及文化、红色、农业、工业、乡村旅游资源，搞好规划布局、产品开发和旅游名镇建设。针对旅游市场需求，加大旅游品牌宣传推介力度，加强行业管理，提高高端旅游设施配套水平，加快构建完整的旅游服务体系。确保全年旅游总收入增长20%。

　　大力发展金融业。规范发展创业投资、工业投资、城建投资等国有投融资平台，增强重大项目投融资能力。积极发展股权投资和风险投资，支持符合条件的企业上市融资。引进境内外金融机构，力争花旗、渣打等银行入驻我市。支持哈尔滨银行做大做强。启动城郊联社改制农村商业银行。继续发展村镇银行、小额贷款公司、融资性担保公司、农村资金互助社等新型金融机构。加快群力、松北金融商务区建设。扩大企业债券发行范围和规模，加快组建城市发展基金，做大做强哈以基金。全年金融业增加值增长30%以上。

　　积极发展物流业。重点推进华南城、双城果蔬绿色有机净菜物流基地等重大物流项目建设。适时启动哈尔滨内陆港异地扩建。积极谋划空港物流园区。大力发展第三方物流，着重引进国内外知名物流企业。研究出台物流业专项扶持意见。确保物流业增加值增长16%以上。

　　着力培育信息服务业。充分发挥专项资金引导作用，加快发展云计算产业，推进哈南“中国云谷”和松北“云飞扬”基地建设。积极推广信息服务在农业、物流、安防、交通以及节能环保等领域的应用。抓好国家“三网融合”试点城市建设和电子商务示范城市创建工作。

　　提档升级会展业和商贸流通业。高质量办好“哈洽会”、“冰雪节”等传统品牌展会和节庆活动，加快建设国际农业博览中心，积极引进国内外知名展会，确保会展业综合收入增长20%以上。优化城市商圈布局，加快改造南岗、道里、道外、香坊等传统商圈，构建松北、哈南、群力、哈西等新商圈。落实国家和省“稳价保供”政策，突出抓好“米袋子”、“菜篮子”，充分发挥价格调节基金作用，努力保障人民群众基本生活。

　　（四）以完善综合服务功能为核心，进一步提升城乡规划建设管理水平

　　突出规划在城乡建设管理中的基础性、先导性作用。编制好城市生态绿道系统、快速公交、历史文化名城保护等专项规划，控详规划、修详规划覆盖中心城区近期重点建设地块，推动产业集聚区与城市功能空间布局规划有效衔接。认真执行《哈尔滨城乡规划条例》，引导城乡经济社会全面协调发展。

　　持续推进现代化基础设施体系建设。重点启动建设王岗、一苗圃高架桥等南部联络线工程，新建改造城市道路87条、桥涵15座，完成保健路、八区地道桥续建工程，大中修一批城区破损道路。继续推进江北百公里滨江景观大道建设，确保呼口大桥建成通车。进一步搞好西客站综合交通枢纽和群力新区公交、学校等配套设施建设。地铁1号线一期力争年底前实现试通车，二期工程和哈西客站地铁联络线建设加快推进。启动花园街保护街区、东西轴线景观大道和空港大道新区联络空间等区域综合改造。改造建设哈亚公路阿城至尚志段等2条国省干线公路，新建续建哈成公路二期等5条城市出口路和京哈高速南沙大街等4个互通立交出口。完成农村公路改造500公里。

　　加强和改进城市常态化管理。管理好城市是政府的重要职责，更是老百姓普遍关注的焦点和热点。没有高水平的管理，就没有高质量的环境。今年我们要开展“城市管理年”活动，全面提高城市管理科学化水平，使市容更整洁、出行更顺畅、景观更宜人。加大市容环境建设管理力度，升级改造旧有居民庭院1027个、社区休闲健身广场20个，新建改造水冲式公厕50座，成立社区物业服务站121个，维修老旧住宅1000万平方米，实现老旧住宅物业管理全覆盖。规范路边停车，新建社会停车场30处，新增停车泊位1.2万个。继续新增大容量绿色环保公交车300台，更新300台，争创国家“公交都市建设示范工程”试点城市。集中整治城乡结合部、城中村、园中村环境以及架空线缆和牌匾广告，综合整治中山路二期、群力新区沿江景观线，高标准做好松花江两岸、湿地岸线及主城区夜景灯饰亮化。加强对磨盘山、西泉眼等城市水源地保护。加快群力西区集中供热等热源项目建设，改造老旧管网276公里，拆并一批小锅炉。开展地下管线隐患排查整改，确保安全运行。健全管理、作业、行政执法联动机制，完善建管交接制度，加快城管“数字化”进程，提高城市管理效能。

　　按照建设国家环保模范城和园林城市目标要求，大力推进生态环境建设。“三沟”治理完成何家沟、马家沟郊区段整治、清水水源工程和信义沟双线截流。阿什河治理完成污水截流和阿城污水处理厂二期工程，启动4个乡（镇）污水处理项目和市区段生态景观建设。松花江干流水质基本达到国家三类标准。松北水网体系开挖渠道45.1公里，新建桥梁30座。推进植树造林和城区绿化，完成人工造林10万亩，新增城区绿地200公顷。启动江北区域固体废弃物综合处理园区建设，推进双琦废弃资源利用示范厂改扩建和餐厨废弃油脂综合处置厂建设。综合整治农村环境，治理水土流失36万亩，创建环境优美乡镇、生态村60个。

　　（五）以创新型城市建设为牵动，进一步增强科技对经济社会发展的支撑引领作用

　　科技创新是调结构、转方式的内生动力。必须抓住科技与经济紧密结合这个根本问题，密切跟踪国际科技和产业发展新动向，加快完善有利于科技创新的体制机制和政策环境。进一步推进技术研发、科技资源信息共享、科技企业孵化、技术转移服务和科技投融资五大科技创新服务平台建设。全年实现技术合同交易额61.6亿元。抓好国家知识产权工作示范城市建设，全年专利申请量增长20%。

　　加速发展高新技术产业。以产业技术联合体为依托，重点抓好发电设备、汽车制造、新媒体等国家级高新技术产业基地建设，加快发展铝镁合金加工、优质动物疫苗等高新技术产业链。鼓励、支持核心技术和重点领域科技研发，全年完成科技攻关项目100个以上。加快光学电流互感器、生物工程及新医药等产业化进程。全年高新技术产业产值增长20%。

　　强化国际科技交流与合作。拓宽交流渠道，完善互访机制，深化合作内涵。高质量办好第四届“哈科会”。加快建设对俄科技合作产学研技术联盟、国际科技合作基地和对俄科技合作中心。抓好“哈以孵化器”建设，促其在哈尔滨与以色列科技合作中发挥示范效应。

　　（六）以开展“项目落实服务年”活动为依托，进一步提高对外开放层次和水平

　　大项目是打基础、攒后劲、惠民生、促发展的关键所在。只有持续不断上项目，才会实现哈尔滨新跨越。加大项目保障服务力度，强化推进措施和谋划手段，确保今年市重点项目开工300项、完成投资1000亿元以上，新谋划3000万元以上项目1000个、亿元以上项目500个。继续跟踪重大经贸活动签约成果，提高合同履约率和项目开工率。

　　扩大招商引资和对外贸易。充分发挥“哈洽会”等经贸平台作用，加强与世界500强等重点企业对接，采取捆绑式招商、产业链招商、“点对点”招商等方式，力争实际使用外资增长10%以上，国内区域经济合作实际到位资金增长20%以上。全面推进与港澳台、欧美、日韩、东盟、以色列等国家和地区的经贸合作，加强与国内发达地区、东北地区经济联系，鼓励有实力的企业开拓国际市场。把握国家沿边开放和俄罗斯远东及西伯利亚地区大开发机遇，加快哈牡绥东对俄贸易加工区哈尔滨段建设，办好对俄出口商品展等展会，支持对俄贸易龙头企业加快发展。

　　坚持把非公有制经济作为经济发展的主体力量。加大扶持力度，落实和完善支持非公经济发展的各项政策措施，降低准入门槛，实行非禁即入，鼓励民间资本进入基础设施、公用事业、金融服务等领域，充分释放民间投资潜力。推进“中小企业成长工程”，重点扶持一批成长性强的企业，新建市级以上中小企业创业基地20个。鼓励扶持微小企业加快发展。

　　（七）以改善民生为根本，进一步拓展惠民的深度和广度

　　解决安居问题，既是群众所盼，也是政府面临的一项长期的、紧迫的、艰巨的任务。必须在注重质量和公平的前提下，加快推进保障性安居工程，确保今年新开工建设保障性住房和棚户区改造住房3.9万套，为1.5万户群众提供廉租住房保障、发放经济适用住房货币补贴,逐步解决城镇低收入群众、新就业职工、农民工住房困难。改造农村泥草房6万户、300万平方米，新建农房480万平方米。切实加强房地产市场监管和调控，确保房地产业平稳健康发展。

　　就业是民生之本。让群众有业可就才能增加百姓收入、促进社会和谐。健全就业服务体系，强化就业培训，做好高校毕业生、农民工、就业困难人员等重点群体就业指导援助，加强农民工劳动保障。全面落实小额贷款担保等扶持政策，鼓励创业带动就业。积极开发公益性服务岗位。全年城镇新增就业10万人以上。

　　提高社会保障和救助水平是保障和改善民生的基本支撑。扩大社会养老保险覆盖面，年底前基本实现新农保和城居保全覆盖，未转非被征地农民应保尽保。扩大医疗保险和新农合受益面，资助低收入家庭重病患者和60周岁以上老人参保参合，城镇居民医疗保险参保率达90%以上，新农合统筹区域内政策性报销比率75%以上。启动城镇居民医疗保险门诊统筹，提高基本医疗保险基金最高支付限额，将残疾人康复治疗纳入城镇基本医疗保险统筹基金支付范围，逐步扩大支付项目。实现工伤、失业保险市级统筹，逐步提高生育保险待遇水平。落实社会救助政策，完善城乡低保标准动态调整机制，确保困难群众基本生活。积极应对老龄化趋势，加快养老体系建设，动员社会力量兴办养老服务事业，新增社会办养老床位1000张。大力发展慈善事业。

　　教育问题事关孩子成长、事关千家万户，老百姓最为关切。必须加大财政对教育投入，尽最大努力办人民满意教育。改善办学条件，新建中小学校4所，创建义务教育标准化学校50所，新改建公办幼儿园78所。实施校舍、校车、校园周边秩序整治工程，为孩子们创造优良学习环境。大力发展职业教育，建立专业化实习实训基地4个，启动建设哈尔滨职业教育园区。推进普通高中多样化、特色化发展，积极办好特殊教育和民族教育，加强高等教育、继续教育和社区教育。建立健全入学和助学机制，切实保障弱势群体受教育权利。

　　让群众方便实惠就医，是医药卫生体制改革的根本目的。我们要切实采取有效措施，解决百姓看病难、看病贵问题。完善医疗卫生服务体系，完成3所市直医院、4所区县医院和10个区县级卫生监督机构建设。从今年开始，市财政连续三年、每年投入5000万元用于市直医疗机构建设。推进非政府办基层医疗卫生机构实施基本药物零差率销售，建立健全基层医疗卫生机构补偿机制。积极稳妥推进公立医院改革，启动医疗纠纷人民调解和医疗责任保险试点。加强疾病预防控制体系建设，提高重大疾病防控和救治能力。加快卫生信息化建设，启动国家级“电子病历”试点，建立城乡居民电子健康档案。继续抓好流动人口计划生育基本公共服务均等化试点。

　　加快发展文化体育事业和产业。推进文化基础设施建设，力争哈尔滨音乐厅和城乡规划展览馆建成并投入使用、哈尔滨大剧院主体封顶、职工文化中心完成配套工程。加快建设平房动漫、印刷出版等6大文化产业园区和冰上舞蹈杂技创作演出等4大基地。深化经营性文化单位转企改制。高水平办好第31届“哈夏”音乐会。落实“全民健身实施计划”，安装健身路径1600件。全力实施八区体育场综合改造工程。

　　（八）以加强和创新社会管理为重点，进一步建设和谐稳定幸福家园

　　社会管理事关社会和谐稳定与长治久安。必须适应经济社会发展新趋势，坚持以人为本、服务为先，推进社会管理创新。切实加强城乡社区和基层自治组织建设，建立社区党组织、居委会与业主委员会、物业协商议事机制，加强社区队伍建设，推行网格化管理，构建新型社区服务管理体系。

　　加强城乡结合部、城中村、新建住宅、流动人口聚集地的居委会组建工作。各区县（市）都要做出具体规划，加快居民公益性服务设施建设，集中建设一批2000平方米以上社区群众文化活动中心。

　　建立综合协调化解社会矛盾的长效机制。完善“大调解”工作体系，强化维稳信息分析和社会矛盾排查机制，从源头上预防和减少矛盾。落实社会稳定风险评估责任制，做到有明显不稳定风险的政策不出台、绝大多数群众不支持的项目不立项。坚持以群众工作统揽信访工作，畅通群众利益诉求表达渠道，全力化解信访积案。充分依靠法治力量和法律手段调处社会矛盾，加强法律援助。

　　加强公共安全管理。构建现代化食品药品安全监管保障体系和综合协调机制，建立健全食品安全追溯体系，加快建设食品安全城市。严抓严管交通、消防、生产安全，坚决遏制重大恶性事故。深入开展“平安哈尔滨”创建活动，创新和完善现代城市治安防控体系，严厉打击各类犯罪。加强网络监管和流动人口管理。强化隐蔽战线斗争。

　　围绕构建社会主义核心价值体系，扎实推进精神文明建设，提高市民文明素质和社会文明程度。深入推进全国文明城市创建工作，开展社会志愿服务主题活动，塑造哈尔滨文明发展的良好形象。在创建文明社区、文明村镇、文明行业、文明单位的同时，组织开展“百家文明单位助百村”共建联建活动，建设一批文明村镇示范点。

　　继续做好广播电视、新闻出版、民族宗教、外事侨务、对台、人防、国防教育和双拥及民兵预备役、统计、档案、地方志、残疾人等工作。

　　加强政府自身建设

　　面对新形势、新目标、新任务和人民群众的新期待，我们必须比以往任何时候都更加注重政府自身建设，更加注重提高政府管理效能和服务水平，更加注重提升政府公信力和执行力，使政府工作符合经济社会发展要求，让人民群众满意。

　　（一）进一步解放思想，创造加快发展的精神动力。坚持把解放思想的着力点放在超越自我上，教育和引导广大公务员认清差距、找准问题，坚决破除一切妨碍发展的落后思想，始终保持积极向上的昂扬斗志和不甘人后的奋斗精神。坚持把解放思想的落脚点放在“忘我”工作上，广泛弘扬路桥精神、环卫精神、交警精神和公仆精神，敢于担当重任、破解难题、突破创新。积极倡导“说了算、定了干、干就干好”的实干精神，以真抓实干、有诺必践的实际行动取信于民、取信于企业、取信于社会，为经济社会发展全局聚合强大动力。

　　（二）切实加强能力建设，不断提高政府行政效能。切实增强抓经济发展的能力，更加注重运用市场手段研究和推动工作，努力掌控产业发展、园区建设、项目招商以及各类经济谈判的主动权。完善吸引、留住和用好人才的各项政策，促进各类人才队伍协调发展。切实增强为群众办实事、解难题的能力，更多地问政于民、问需于民、问计于民，努力把群众的根本利益维护好、实现好、发展好。切实增强处理复杂问题的能力，进一步完善应急管理机制，做到突发公共事件及时发现、快速应对、妥善处置。切实强化绩效考核和行政问责，提高行政效率和服务水平。

　　（三）全面推进依法行政，努力建设法治型政府。自觉接受人大依法监督、政协民主监督和社会监督、舆论监督。支持工会、共青团、妇联等群团组织开展工作。全面贯彻落实“六五”普法规划，提高广大干部群众法律素质。规范行政执法行为，严格执法程序，强化执法监督。加大行政复议等领域改革力度，提高政府化解行政争议能力。

　　（四）着力提升服务质量，切实改善经济发展环境。坚持把政风建设作为优化环境的着力点和突破口，不断强化“企业创造财富、政府创造环境”理念，巩固和扩大“政风建设年”成果。继续深化行政审批制度改革，全面实行企业行政审批“一表制”，推行“一口受理、抄告相关、同步审批、协同会审、联合踏勘、统一收费、限时办结”审批模式，全面提高行政审批服务水平。

　　（五）扎实推进廉政建设，树立清正廉洁政府形象。认真落实党风廉政责任制，深入推进惩治和预防腐败体系建设。强化公务员教育和权力运行的制约监督，完善反腐倡廉制度，逐步形成用制度管权、管事、管人的工作格局，确保权力在阳光下运行。坚决查处各类违法违纪案件，始终保持惩治腐败高压态势。

　　各位代表！坚定实施新战略，奋力实现科学发展新跨越，目标宏伟、任务艰巨。让我们更加紧密地团结在以胡锦涛同志为总书记的党中央周围，在省委、省政府和市委领导下，紧紧依靠全市人民，齐心协力，奋发有为，开拓创新，推进全市经济社会更好更快发展，以优异成绩迎接党的十八大胜利召开！
